# Implementation of Physical Activity in the Curricula of Medical Schools and Healthcare Professions Across Europe: The VANGUARD Project Study Protocol

**DOI:** 10.31138/mjr.290724.iac

**Published:** 2024-09-30

**Authors:** George S. Metsios, Ioannis D. Morres, Ioannis Fatouros, Ian M. Lahart, Ramune Zilinskiene, Natalja Istomina, Rytis Jankauskas, Jarek Maestu, Katerina Tzika, Eve Unt, Romeu Mendes, Ana Barbosa, Anne Vuillemin, David Darmon, Ann B. Gates

**Affiliations:** 1 School of Physical Education, Sport Science and Dietetics, Department of Nutrition and Dietetics, University of Thessaly, Greece; 2 School of Physical Education, Sport Science and Dietetics, Department of Physical Education and Sports Science, University of Thessaly, Greece; 3 Faculty of Education Health & Wellbeing, University of Wolverhampton, United Kingdom; 4 Vilniaus universiteto Sveikatos ir sporto centras/Vilnius University Health and Sports Centre, Lithuania; 5 Medical Faculty of Vilnius University, Sveikatos mokslų institutas/Institute of Health Sciences, Lithuania; 6 Institute of Sport Sciences and Physiotherapy, University of Tartu, Tartu, Estonia; 7 Institute of Clinical Medicine, University of Tartu, Tartu, Estonia; 8 EPIUnit-Instituto de Saúde Pública, Universidade do Porto, Porto, Portugal; 9 Unidade Local de Sáude de Trás-os-Montes e Alto Douro, Vila Real, Portugal; 10 Université Côte d’Azur, LAMHESS, Nice, France; 11 Université Côte d’Azur, RETINES, Nice, France; 12 School of Health Sciences, The University of Nottingham, Nottingham, United Kingdom

**Keywords:** physical activity, implementation, medical education, non-communicable diseases, health

## Abstract

**Introduction:**

The 2018 published World Health Organisation (WHO) Europe physical activity factsheet reports, specify agreed targets for physical activity and articulate the need to improve the education of medical doctors and healthcare practitioners in order to increase physical activity and reduce sedentary time in people at risk and/or living with Noncommunicable Diseases (NCDs). Given the dearth of relevant initiatives and the continuous need to increase physical activity participation towards better health management of NCDs, the aim of this study is to embed physical activity in the undergraduate curricula of future frontline healthcare professionals (medical doctors and allied health professions) in European countries.

**Methods:**

The **V**irtual **A**dvice, **N**urturing, **G**uidance on **U**niversal **A**ction, **R**esearch and **D**evelopment for physical activity and sport engagement (VANGUARD) project consists of a collaborative partnership Consortium between six European Universities, WHO Europe and Ministry representatives that has been developed to implement physical activity in the curricula of medical schools and healthcare professions. The methodology of the VANGUARD project is informed by the WHO implementation guidance and the Reach, Effectiveness, Adoption, Implementation and Maintenance (RE-AIM) framework.

**Discussion:**

Through a carefully planned implementation process and via using established appropriate implementation evaluation tools, the end result of the VANGUARD project will be the a) implementation of a physical activity module in six different European Universities (five medical schools and one physiotherapy department) and b) development of a toolkit/guide, in order to assist other healthcare systems and European Universities to develop relevant grass-root innovations for addressing the decline in physical activity levels.

## INTRODUCTION

Physical activity is any movement of the human body and skeletal muscle that increases energy expenditure; exercise on the other hand, is a subset of physical activity and is a planned, structured and repetitive activity that is intended to improve or maintain specific physiological capabilities and outcomes. Increasing physical activity is a behaviour that has beneficial effects in multiple symptoms and clinical outcomes across different non-communicable diseases (NCDs). For example, collective research evidence suggests physical activity can significantly benefit major public health challenges. For example, physical activity can alleviate depressive symptomatology,^1^ reduce cardiovascular mortality and improve symptoms of cardiovascular disease,^2^ quality of life and specific symptoms in cancer,^3,4^ and autoimmune inflammatory disease,^5,6^ while at the same time reduce the cost for European (EU) healthcare.^7^ As such, physical activity has been identified as a best-buy intervention by the World Health Organisation (WHO) to prevent NCDs as well as to manage and promote health and wellbeing.^8^ Despite the benefits of physical activity on NCDs, the world population, particularly in the higher income countries, is becoming less active. This is evident from the comparison of the physical activity Eurobarometer data between 2014 and 2018,^9^ showing an increase from 42% to 46% in the proportion of European citizens that never exercise or play sports. Unfortunately, this is a continuation of a gradual decreasing trend in physical activity engagement seen from 2009. Therefore and evidently, the significant efforts to increase physical activity and decrease sedentary behaviour are failing or are not effective to achieve the set public health targets.

The 2018 published WHO Europe physical activity factsheet reports, specify agreed targets for physical activity and articulate the need to improve the education of medical doctors and healthcare practitioners in order to increase physical activity and reduce sedentary time in people at risk and/or living with NCDs.^13^ The WHO Europe has made a considerable effort to collect baseline data from all EU medical and healthcare professional academic entities in terms of physical activity education of undergraduate medical and healthcare professionals. WHO Europe concludes^8,11^ that a universal and systematic approach for the education of undergraduate future frontline health practitioners with tailored, peer-reviewed, and evidence-based resources, is missing from many national strategies. This shortcoming may have detrimental effects on NCD patients’ management and healthcare system cost, as the clinical practice and management of NCDs is not optimal nor are opportunities to promote physical activity and sport participation being recognised. This also means that the education and health systems are not adequately developing the required leadership capacity in the health workforce to change and implement physical activity policy. Leadership within the academic community and clinical practice and change agency implementation by educators may be effective interventions that are likely to play important roles in the adoption of sustainable and successful knowledge/skills in the future workforce of healthcare practitioners.^12^ Moreover, through their education, medical and healthcare students can start – via their curriculum/education – to develop their leadership skills and actions within their sectors (healthcare services) to empower their individual clinical practice regarding physical activity, and lead the strategic change that is required within the system delivery models outlined by the WHO.^13,14^ Given the dearth of relevant initiatives and the continuous need to improve physical activity participation for promoting health and better manage NCDs, the aim of the present study is to embed physical activity in the undergraduate curricula of future frontline healthcare professionals (medical doctors and allied health professions) in European countries.

## METHODS/DESIGN

This manuscript adheres to the Standards for Reporting Implementation Studies (StaRI) Statement.^15^

### Study team

The Virtual Advice, Nurturing, Guidance on Universal Action, Research and Development for physical activity and sport engagement (VANGUARD) project consists of a collaborative partnership Consortium between six European Universities, WHO Europe and Ministry representatives that has been developed to implement physical activity in the curricula of medical schools and healthcare professions. The partners of the project are the a) University of Thessaly, Greece (coordinator), b) University of Porto, Portugal, c) University of Tartu, Estonia, d) University of Vilnius, Lithuania, e) University of Cote d’Azur, France and f) University of Wolverhampton, UK. The study team includes an interdisciplinary group of strategists, academics, researchers, clinicians, university administrators and student representatives with expertise in implementation science, education, physical activity, biostatistics, mixed methods, and NCD clinical care.

### Implementation approach and framework

The methodology of the VANGUARD project is informed by the WHO implementation guidance^16^ and the Reach, Effectiveness, Adoption, Implementation, and Maintenance (RE-AIM) framework. The RE-AIM framework is widely used and accepted in healthcare sciences, and can evaluate outcomes relevant to translating research into practice.^17^ The RE-AIM framework has been used in diverse settings to assess real world performance of evidence-based initiatives^18^ but it is also utilised to pragmatically match evaluation needs.^19^ The combination and utilisation of both these methodologies (WHO and REAIM), ensure a step-by-step and evidence-based approach for effective implementation.

### Setting

The present study will be conducted in five different European countries and the UK, each having different contextual factors that may affect implementation, as well as different national priorities and initiatives in terms of physical activity adoption, albeit all align with the European and WHO physical activity strategies. All VANGUARD partners will implement a physical activity module in the curricula of their respective medical schools, while the University of Wolverhampton will implement physical activity in the curriculum of the Physiotherapy course (i.e. healthcare profession). The reason for this approach is because 12 out of 16 medical schools in the UK, have already implemented physical activity in their curricula, as part of a relevant previous project in the UK.^14^ In addition to that and following implementation science principles, the University of Vilnius, Lithuania, will also implement the physical activity module in another five healthcare practitioner and medical colleges and universities in Lithuania, as a part of a VANGUARD scaling-up exercise. In specific, The University of Vilnius will help implement a physical activity module in a) the Lithuanian University of Health Sciences, b) the Klaipeda University, c) the Vilnius College, d) the Siauliai State College and e) the Utena College.

### Implementation methodology

#### Step 1: Implementation intelligence and development of the implementation map

In each of the six collaborating Universities, the national VANGUARD academics will lead on co-developing an implementation methodology, which will be tailored to each University’s context and specificities (using the WHO implementation guide and the RE-AIM Implementation Framework). To achieve this, focus groups with key stakeholders will take place in each collaborating University, and specifically a) higher management (Rector, Vice Rector, Faculty higher management, such as Dean), b) academic and c) student representatives. The outcomes of these focus groups will be to: a) agree on and co-develop a clear framework of embedding physical activity in the curriculum that recognises the University-specific differing levels of engagement and support, b) provide scene setting evidence-based, up-to-date, peer-reviewed presentations and infographics that promote WHO-, EU-, country- and University-specific recommendations for physical activity curriculum change (e.g. #MovementForMovement resources),^20^ c) enable discussions for country-specific tailored approaches on how to deliver physical activity in the curriculum d), establish a baseline of existing physical activity teaching and assessment, e) plans and actions to identify specific examples/academics of best practice and innovation in physical activity leadership, teaching and assessment methods. It is important to note that all participating Universities will follow the above same Step 1 implementation methodology, accepting however, that the actual implementation process may be slightly different in each country (i.e. some Universities may decide to implement compulsory and some others elective modules, depending on what the stakeholder focus groups will decide and conclude in each University). This organic process is necessary in implementation science in order to tailor implementation according to identified barriers and facilitators which are specific and unique in each setting.^16^

#### Steps 2 and 3: Implementation of physical activity in curricula

This step will initiate the implementation of the new physical activity module in the curricula of undergraduate doctors and healthcare practitioners.

The material for the lectures will be based on country-specific Step 1 implementation intelligence, WHO/EU reports for physical activity/sport and national and international strategies, and blended learning (e.g. focus groups, interactive learning, videos), all tailored to each University’s needs, guidance and processes as well as preferences (identified in Step 1). The materials for teaching will also rely on the #MovementForMovement resources, which will be bespoke to each University. The overall implementation and delivery process will be overseen by the national VANGUARD academic lead. A team of academics from each country will also be collectively responsible for monitoring and evaluation of the implementation outcomes and specifically for recording the progress, attendance, assessment arrangements and assessment rates in comparison to other taught modules (as per standard practice) as well as student feedback. After the initial implementation of the physical activity modules, further issues that have not been identified in the co-development phase, will be resolved with additional capacity building (e.g. lack of attendance, reduced student satisfaction).

**Figure 1.**
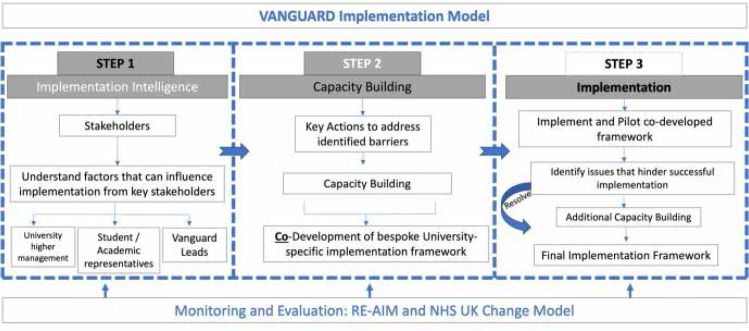
VANGUARD Implementation methodology.

#### Study timeline

The VANGUARD project started in January 2020 and is funded by the ERASMUS Sport + initiative (project number: 613494-EPP-1-2019-1-UK-SPO-SCP). Despite the fact that initially the VANGUARD project was developed to last for three years, due to the COVID-19 pandemic, the project has received a one-year extension by the European Commission, and thus, will now last four years (project ends in 2024). The three first years of the project were dedicated to Step 1, while the last year for Step 2. The overall project evaluation will take place after the last year of the project, when all VANGUARD partners will have implemented and evaluated the physical activity module within their curriculum.

#### Implementation evaluation

The primary aim of the study is implementation fidelity, i.e. if the six VANGUARD Universities will indeed implement the physical activity module within their curriculum. In addition to the primary outcome, the VANGUARD Consortium will utilise two different tools to evaluate the whole implementation process (secondary outcomes):

#### a) RE-AIM scoring instrument

The RE-AIM scoring instrument has different dimensions/components that can be used to evaluate interventions, such as the one described herein. Although this tool has been predominantly developed to evaluate healthcare interventions, its proposed dimensions are relevant to the primary and secondary outcomes on the VANGUARD project. **Table 1** depicts the VANGUARD RE-AIM components, measures and key performance indicators.

**Table 1.** RE-AIM components, measures and key performance indicators.

Components: program goals	Measures: project outcomes
Reach	Student a) interest, b) barriers and facilitators, c) suggestions for future improvement
Effectiveness	Effectiveness of teaching, knowledge, usefulness of module resources, student satisfaction with the module knowledge and teaching style
Adoption	Personal time studying on the subject matter outside the module time, downloading and using the module resources
Implementation	Implementation efficacy i.e. if the module was finally implemented or not in the curriculum
Maintenance	Curriculum change

#### b) United Kingdom National Health Service implementation model for change

To provide a significantly better depth in the data obtained, the VANGUARD project will also utilise the United Kingdom National Health Service implementation model for change. This can help record and evaluate significant implementation processes but also other important components that can contribute to long-term sustainability and change. Briefly, the Health Service implementation model is based on eight components, and specifically:

1. Shared purpose (critical driver of success in organisational performance and change), which will be based on the evaluation of three distinct steps (create a safe space, identify commonalities and co-develop the implementation).
2. Spread and adoption (achievements, good practice, tips and learning from what worked and what didn’t), that will be evaluated by the NHS Institute’s spread and adoption tool, as described in the Health Service implementation model.
3. Improvement tools (other components of the Change Model and provide an underlying structure for change efforts) that will be evaluated by the Five Step Approach model.
4. Project and Performance Management (creation of a clear plan and an ongoing review of its actions, targets and milestones) that will be evaluated by the Rigorous Delivery Framework.
5. Measurement of the outcome of change, that ensures that risks are mitigated, effective change is happening, and results are being achieved. As part of this step we will also evaluate: a) how many courses accessed the resources, b) how many students attended class, c) how many students in total accessed the resources per year as well as throughout the lifetime of the course, d) attendance to the module, e) student satisfaction, f) student feedback on the resources and the whole course, g) staff satisfaction for the use of the resources, h) method of assessment and pass rate.
6. System Drivers (e.g. personal incentives, organisational or regional or national drivers) will be evaluated by reporting the key intrinsic and extrinsic motivators.
7. Motivation and Mobilisation (engagement of key stakeholders for co-developing effective system changes), which will be evaluated by reporting the 5 Energies of Change, and
8. Leadership by all that will be evaluated via describing the approach, skills and behaviours needed for the intended change. The nine dimensions of the Health Leadership model will be used for this evaluation.

#### Governance

To ensure the timely delivery of the project outcomes, the VANGUARD project has set interim meetings every six months as well as yearly meetings throughout the project lifetime. These platforms will serve as a means of risk identification and stratification but also as a platform to share good practice methods from all partners, a method that may provide solutions to partners facing different implementation difficulties (e.g. slow adoption, difficulty in implementation or slow progress due to the COVID-19 pandemic). Moreover, each country will identify a project country lead that is responsible for the realisation of the study aims and deliverables in a timely manner, as well as to collect country specific data and communicate them with the wider VANGUARD team.

## DISCUSSION

The VANGUARD is the first European project that has been designed to implement physical activity modules in medical schools and healthcare professional curricula, in order to help address the continuing decline in physical activity levels. We anticipate that this will be achieved, via knowledge development within the current medical and healthcare curricula, so that current students and thus, future doctors and healthcare practitioners, can use physical activity in their clinical practice. Via the utilisation of evidence-based approaches, University education is a means of developing, the capacity and analytical skills of the future workforce to improve different aspects of today’s societies. In a continuous changing EU environment, a significant contemporary challenge is to help stop the decline of the physical activity levels of European citizens. As such, the VANGUARD project was designed to be in line with the implementation of the WHO Global Action Plan for Physical Activity (GAPPA 2018–2030)^13^ as well as with all four objectives of the WHO GAPPA 2018 report; it also contributes to the delivery of specific Sustainable Development Goals (SDGs).^21^ All these reports suggest the use of capacity building to upskill the European workforce in order to address major challenges, such as the lack of physical activity. Moreover, the project addresses existing European Policies that aim to strengthen European Cooperation for the promotion of healthier lifestyles, such as the “Tartu call for a healthy lifestyle”.^22^

Increasing physical activity is a behaviour that associates with significant health benefits in different NCDs, as evidenced via different systematic reviews and meta-analyses.^2,3,23^ Moreover, physical inactivity significantly contributes to the increasing costs of healthcare services worldwide. An analysis of the costs of physical inactivity in 142 countries (93.2% of the world’s population) suggests that it can equate to 53.8 billion American dollars per year.^7^ As such, addressing this important societal challenge has become one of the main priorities of the leading healthcare societies. The VANGUARD project will help address this, via educating current student doctors and healthcare practitioners about the beneficial effects of physical activity in different NCDs, so that they can become more knowledgeable about the beneficial effects of physical activity in NCDs. As such, although the main outcome of the VANGUARD project is to educate doctors of six different EU Universities about the benefits of physical activity in NCDs via implementing a relevant module in the curriculum, the anticipated long-term outcome of this project is for future healthcare practitioners to be able to advise physical activity to their NCD patients. However, the timeline of the VANGUARD project (four years) does not allow for evaluating whether indeed, these doctors and healthcare practitioners will promote physical activity in their clinical practice, as this would require a significant observation time. However, advising NCD patients to increase their physical activity levels can potentially be a significant advancement in future clinical practice, since literature findings suggest that NCD patients want their “trusted managing doctors and healthcare practitioners” to act as a trigger for them to change behaviours and be more physically active.^24^ Through a carefully planned implementation process and via using established appropriate implementation evaluation tools, the VANGUARD project will report its findings in a timely manner. The end result of the present project will be the development of a toolkit/guide, in order to assist other healthcare systems and European Universities in developing relevant grass-root innovations for addressing the decline in physical activity levels.
